# LSTMAtU-Net: A Precipitation Nowcasting Model Based on ECSA Module

**DOI:** 10.3390/s23135785

**Published:** 2023-06-21

**Authors:** Huantong Geng, Xiaoyan Ge, Boyang Xie, Jinzhong Min, Xiaoran Zhuang

**Affiliations:** 1 School of Computer, Nanjing University of Information Science and Technology, Nanjing 210044, China; htgeng@nuist.edu.cn (H.G.); 20211221043@nuist.edu.cn (B.X.); 2 Jiangsu Open University, Nanjing 210036, China; 3 School of Meteorology, Nanjing University of Information Science and Technology, Nanjing 210044, China; jzmin@nuist.edu.cn; 4 Jiangsu Meteorological Observatory, Nanjing 210008, China; zxrxz3212009@163.com

**Keywords:** precipitation nowcasting, U-Net architecture, Convolutional LSTM (ConvLSTM), Efficient Channel and Space Attention (ECSA)

## Abstract

Precipitation nowcasting refers to the use of specific meteorological elements to predict precipitation in the next 0–2 h. Existing methods use radar echo maps and the Z–R relationship to directly predict future rainfall rates through deep learning methods, which are not physically constrained, but suffer from severe loss of predicted image details. This paper proposes a new model framework to effectively solve this problem, namely LSTMAtU-Net. It is based on the U-Net architecture, equipped with a Convolutional LSTM (ConvLSTM) unit with the vertical flow direction and depthwise-separable convolution, and we propose a new component, the Efficient Channel and Space Attention (ECSA) module. The ConvLSTM unit with the vertical flow direction memorizes temporal changes by extracting features from different levels of the convolutional layers, while the ECSA module innovatively integrates different structural information of each layer of U-Net into the channelwise attention mechanism to learn channel and spatial information, thereby enhancing attention to the details of precipitation images. The experimental results showed that the performance of the model on the test dataset was better than other examined models and improved the accuracy of medium- and high-intensity precipitation nowcasting.

## 1. Introduction

Severe convective weather occurs frequently in China, and short-term heavy precipitation causes other disaster chain reactions, which cause serious economic losses and casualties. Precipitation nowcasting can make an early warning plan for this weather and reserve a time window for disaster prevention and mitigation [1,2]. It can also ensure the correct operation of emergency services, energy management, maritime services, and other industries. Numerical Weather Prediction (NWP) [3] is a commonly used precipitation forecasting method that uses numerical calculations to solve physical equations to predict precipitation in a certain period of time in the future. However, the nowcasting performance of NWP is poor because the forecast period of nowcasting is less than the time required for the model to reach steady-state simulation [4]. It relies on many computational data, which requires high-performance computers. In order to solve these problems, radar-based precipitation nowcasting has become an important development direction.

**Figure 1:**
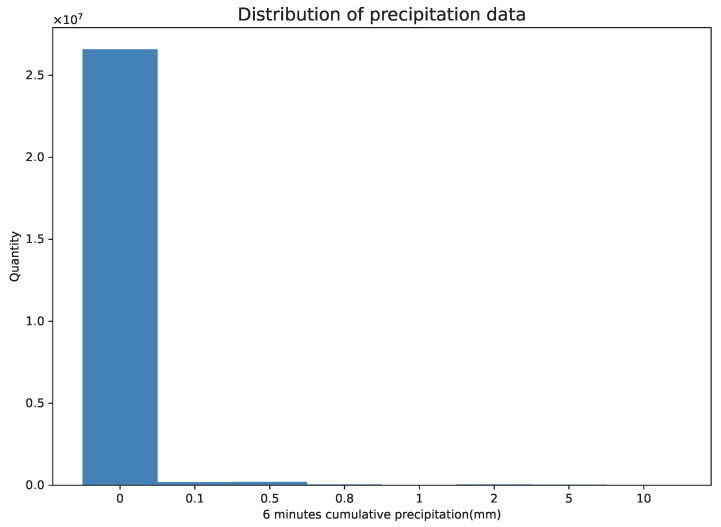
Precipitation data distribution statistics. The data were derived from several random sequences of the precipitation dataset in the 2022 Jiangsu Meteorological AI Algorithm Challenge. The spatial resolution of the single-time data is 480 × 560; the time period is 6 min; the total sample number is 101.

Since Ligda [5] proposed the precipitation nowcasting based on radar elements, this method has been widely recognized and deeply studied [6,7,8,9,10,11], so it has become the mainstream forecasting technology for heavy precipitation nowcasting. It first uses the radar echo extrapolation model to predict the future radar echo and then uses the Z–R relationship [12] to obtain precipitation data. However, predicting in these two steps will lead to cumulative error, while precipitation nowcasting based on precipitation elements or fusion elements [13,14,15,16,17,18,19] will reduce this error. It directly uses several precipitation frames or fusion feature frames to predict future precipitation maps. However, the precipitation data used in this method have the following problems: First, due to the very few to no rain samples in the precipitation data, there is the problem of an uneven distribution of data samples and a deviation of the data grade distribution in the precipitation dataset, as shown in Figure 1. Second, precipitation data have the characteristics of high-order nonlinearity and an extremely complex spatial and temporal distribution [20]. This makes it difficult to model with a specific inflexible target.

Deep learning methods based on precipitation features’ or fusion features’ extrapolation include Recurrent Neural Networks (RNNs) and Convolutional Neural Networks (CNNs). RNN-based models have strong modeling capabilities for time series data, which can be used to mine the spatiotemporal evolution of precipitation sequences [13,14], but they cannot solve the problem through parallel computing, prolong the prediction time, and have the problem of gradient explosion. CNN-based models not only have a strong feature extraction ability, but also can shorten prediction time by parallel computing. Among them, the authors in [21] introduced the famous U-Net model, which was originally used for medical image segmentation, but also has had success in precipitation nowcasting [15,16,17,18,19]. However, it has limited long-term dependency capabilities due to the inherent local nature of the convolutional operation. In addition, the models based on the RNN and CNN frameworks both have the problem of gradually inaccurate details in a long delivery cycle.

Motivated by this, this paper proposes LSTMAtU-Net, a deep learning network based on precipitation features’ or fusion features’ extrapolation. It is based on the U-Net architecture, equipped with a ConvLSTM [10] unit with the vertical flow direction, the ECSA module, and DSC [22]. The framework significantly improves the accuracy of precipitation nowcasting. Here, we used the dataset of the 2022 Jiangsu Meteorological AI Algorithm Challenge to evaluate the framework, which predicts the structured data of precipitation elements in the next two hours based on the weather radar images and the structured data of meteorological elements that have occurred in the past.

In summary, the main contributions of this paper are as follows:

1. The LSTMAtU-Net model combines the advantages of RNNs and CNNs. We propose a model that combines the RNN and CNN structures, namely the LSTMAtU-Net model. It has a strong feature extraction capability and improved long-term dependency capabilities, and the core convolutional structure greatly reduces the risk of gradient explosion.
2. A new component, the ECSA module, is proposed, which uses mean pooling at different scales as a way to achieve the weighting of the channel and spatial information of convolutional features based on the ECA module [23]; thus, focusing on image details alleviates, to a certain extent, the problem of the RNN’s and CNN’s predictions showing the gradual inaccuracy of the image details.
3. We propose a completely new loss function. It first optimizes multiple prediction objectives and then designs the corresponding weighted loss function to solve the serious imbalance of precipitation data.

The rest of the paper is organized as follows: Section 2 briefly introduces the related work on nowcasting. Section 3 describes our proposed model. Comprehensive experiments that verified the effectiveness of our model are given in Section 4. The conclusion is given in Section 5.

## 2. Related Works

In this section, we will introduce deep learning models for nowcasting. Since our model also includes an attention mechanism, we also introduce the related research work on attention mechanisms.

### 2.1. Nowcasting Models Based on ConvRNN

ConvLSTM [10] was the first precipitation nowcasting model based on the ConvRNN, which adds convolutional layers to FC-LSTM, giving the network the ability to extract spatial information on top of being able to extract temporal information very well. The authors of [24] considered [10] the uneven extraction of temporal and spatial information and proposed the PredRNN model, which adds a unit storing propagation spatial information in each LSTM module to enable the model to propagate longitudinally. Continuing the study of [24], the authors of [25] proposed the PredRNN++, which adds the Gradient Highway Unit (GHU) to the PredRNN to deal with the gradient disappearance problem. To solve the difficulty of predicting higher-order nonsmooth information in radar echo maps, the authors of [26] proposed the MIM mechanism, which can effectively extract nonsmooth information. RN-Net [14], which fuses the spatiotemporal characteristics of rainfall data and radar data, provides a more adequate basis for forecasting. Based on the research of [14], the same team [13] proposed the MFSP-Net model, which uses meteorological data with different spatiotemporal resolutions as the input and combines spatiotemporal prediction and multi-model fusion in depth with a multi-task learning strategy. GAN-rcLSTM [11] introduced the Generative Adversarial Network (GAN) technology. The discriminator consists of several 3D convolutions and a fully connected layer. The generator is the proposed residual LSTM module, which can reduce the gradient disappearance problem and avoid the weakness problem of deep LSTM extrapolation to a certain extent. In addition, it makes the extrapolation sequence clearer through the generator and discriminator.

### 2.2. Nowcasting Models Based on U-Net

U-Net [21] is a classical fully convolutional network, which consists of a contracting path and a symmetric expanding path. The contracting path is used to obtain context information, and the expanding path is used for accurate positioning. ResU-Net [19] introduced the attention mechanism to the ResNetof [21], where the attention mechanism module was incorporated to learn temporal information. Broad-U-Net [18] has asymmetric parallel convolutions and an Atrous Spatial Pyramid Pooling (ASPP) module by which it combines multiscale features to learn more complex patterns. SmaAt-U-Net [15] reduces the network size significantly, but the prediction performance is comparable to other models. Based on [15], the authors of [16] proposed AA-TransU-Net, which introduces the Transformer mechanism into precipitation nowcasting with some improvement in forecast accuracy.

### 2.3. Attention Mechanism

The core idea of the attention mechanism is to find correlations among the original data and then highlight some of their important features, and some studies in precipitation nowcasting have introduced the attention mechanism to precipitation nowcasting. The Squeeze-And-Extraction Network (SENet) [27], which belongs to the family of channel attention mechanisms, is very simple to construct and does not require the introduction of new functions or layers. Besides, it has good properties in terms of model and computational complexity. The Convolutional Block Attention Module (CBAM) [28] is a fusion of spatial and channel attention mechanisms, which can learn the importance of the channel and space separately and also can be easily embedded into any known framework. The Efficient Channel Attention (ECA) module [23] solves the side effects caused by the dimensionality reduction in SENet and achieves good results using only a few parameters.

The ConvRNN-based nowcasting model, developed from ConvLSTM and GAN-rcLSTM, is dedicated to solving the effective transmission of spatiotemporal information, the problem of gradient disappearance, and the ambiguity of prediction. Although the accuracy of precipitation was improved to a certain extent, it still has the problem that it cannot be parallelized, which will prolong the prediction time and produce low-quality prediction results due to the stochastic dynamic environment of precipitation [29,30]. The U-Net-based nowcasting models developed from U-Net and AA-TransU-Net are all devoted to solving the problem of the limited ability of the convolutional model to model long-range dependencies [29]. Although the ability to learn the time factor gradually becomes stronger, the effect is still not good in a long delivery cycle. Therefore, we combined these two structures to improve the long-term dependence capability, shorten the prediction time, and capture the complex spatial information to produce better prediction results. In addition, in order to better focus on image details, we introduced an attention mechanism, using the ECSA module. This is an improved version of the ECA module [23]. The existing ECA module has the problem of losing much of the spatial structure information, because it contains a Global Average Pooling (GAP) module function and the feature map size is generally 7 × 7 [31], while the U-Net architecture contains feature maps of different sizes. Therefore, based on this motivation and the idea of [31], the ECSA module adapted to different scales was established.

## 3. Methodology

### 3.1. Problem Definition

The problem of precipitation nowcasting based on precipitation features or fusion features can be approximated as a sequence prediction problem. The input and predicted data in this task are both structured data sequences of precipitation elements. Consider *X* as the input data, which is a series of three-dimensional tensors in time period *M*, and $\hat{Y}$ as the predicted outcome, which is a sequence of data in time period *N*. $X\in R^{H\times W\times C_{in}}$, $\hat{Y}\in R^{H\times W\times C_{out}}$, where H,W is the spatial resolution of the image, $C_{in}$ is the number of input data channels multiplied by the time period *M*, and $C_{out}$ is the number of predicted data channels multiplied by the time period *N*. This is due to the need to map the time dimension to the channel dimension. *F* is the proposed model LSTMAtU-Net, then the whole prediction process is defined as:

(1) $$\hat{Y}=F\left(X\right)$$

### 3.2. LSTMAtU-Net

**Figure 2:**
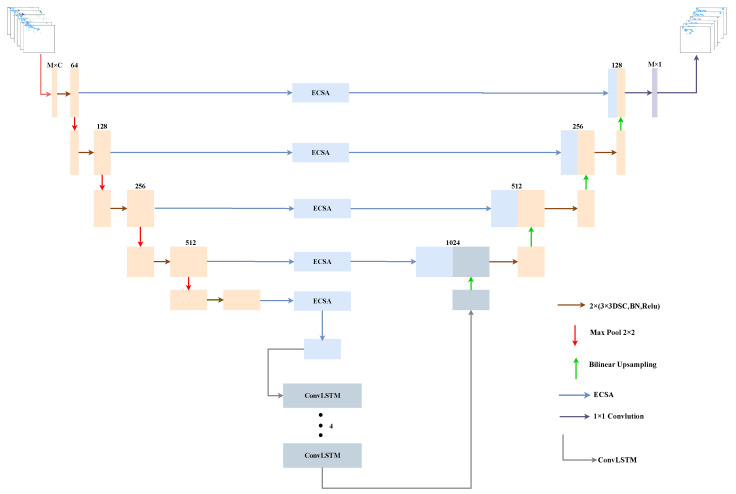
LSTMAtU-Net model framework. It contains the encoder–decoder structure. Each bar in the figure represents a multi-channel feature map, and the number represents the number of channels. The input is an example of precipitation, and the output is the corresponding prediction map generated by the LSTMAtU-Net model. On the right side of the diagram is the description of each arrow.

The model proposed in this paper is based on the U-Net architecture. The model first replaces the convolutional part of U-Net with DSC. Next, the ECSA module and the ConvLSTM unit with the vertical flow direction are placed in the skip-connection of the encoder and decoder. The importance weights of each feature of the generated image are derived by the former, and the latter extracts and memorizes the spatiotemporal representations to learn the importance of the temporal factor. Finally, the functions of the model’s encoder and decoder are consistent with those of U-Net, with the encoder responsible for feature extraction and the decoder responsible for restoring the image to its original size, resulting in a pixel-by-pixel prediction. The model framework is shown in Figure 2. The settings of the DSC and ECSA modules reduce the number of parameters in order to have better performance. In Table 1, we compare the LSTMAtU-Net model with the ECSA module and SENet regarding the number of parameters of our model. Our model reduces the model parameters by about 41.86 % compared to the model without these two modules.

Encoder: As seen in the left half of Figure 2, this starts with two convolutional layers to process the input image into a feature map suitable for the encoder. This is followed by four consecutive modules, which contain a Max pooling layer (red arrow in Figure 2) and a double convolutional layer (each convolutional layer includes a 3 × 3 DSC, Batch-Norm (BN) [32] layer, and Rectified Linear Unit (ReLU) [33]) (brown arrow in Figure 2). The featured image obtains a larger field of view and more essential features as the network layers go deeper.

Decoder: As seen in the right half of Figure 2, this starts with the output of the ConvLSTM unit, followed by 4 consecutive modules, differing from the encoder’s module in that it contains a bilinear upsampling layer (green arrow in Figure 2) and connects the upsampled feature mapping of doubled the size to the feature mapping output from the previous ECSA module. Finally, the predicted image is output with a 1 × 1 convolutional layer (purple arrow in Figure 2).

Skip-connection section: This includes the ECSA module and ConvLSTM unit with the vertical flow direction.

**Figure 3:**
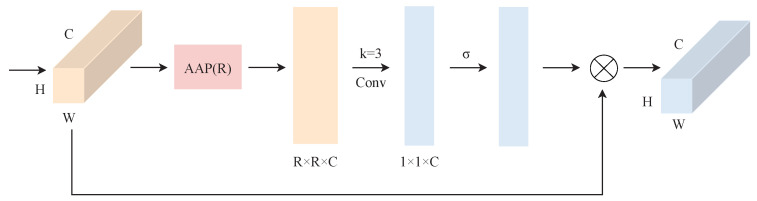
ECSA module architecture diagram. R denotes for each layer different feature map sizes adapted to the output of different scales; for example, R in the first layer of the ECSA module is taken as 16. AAP is the Adaptive Average Pooling layer.

The channel attention mechanism generally uses GAP to aggregate global information in each channel, while the U-Net architecture outputs different sizes of feature maps in each layer, which leads to a large amount of lost spatial information. To solve this problem, we propose the ECSA module, which is a local cross-channel interaction module without dimensionality reduction, with the structure shown in Figure 3, where the convolutional kernel size is experimentally shown to be the best with a size of 3.

Given a feature map $X\in R^{C\times H\times W}$ and denoting the adaptive average pooling layer as $P\left(\cdot ,\cdot \right)$, $C1D_{3}\left(\cdot \right)$ as a 1 × 3 one-dimensional convolution, and $\sigma \left(\cdot \right)$ as the Sigmoid activation function, then the output $\tilde{X}$ of the ECSA module can be represented as shown in (2).

(2) $$\tilde{X}=\sigma \left(C1D_{3}\left(P\left(X,R\right)\right)\right)⊗X$$

It first adaptively outputs features of different scales R (16 × 16 for the first layer, 8 × 8 for the second layer, 4 × 4 for the third layer, 2 × 2 for the fourth layer, and 1 × 1 for the fifth layer) according to the different feature map dimensions of each layer to obtain the spatial structure information of R × R × C. Then, the feature compression is performed along the spatial dimension, and each feature channel is transformed into R × R real numbers; then, this information is convolved to learn the importance of different channel features, and at this time, a feature map of dimension 1 × 1 × C is output. Finally, the output feature map is multiplied by the original input feature map channel, so that the spatial structure information and channel information can be integrated simultaneously to improve the attention to the precipitation area.

**Figure 4:**
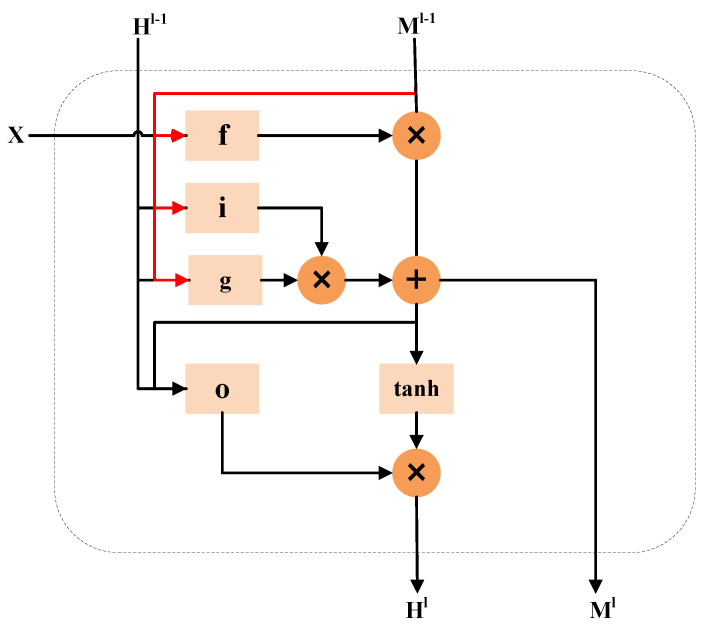
The structure of the ConvLSTM unit with the vertical flow direction, where f is the forgetting gate, i and g are the memory gates, and o is the output gate.

The detailed structure of the ConvLSTM unit with the vertical flow direction is shown in Figure 4. It is built with convolution to combine the CNN layer with the LSTM cell in order to grasp both the spatial and temporal information. This structure is equivalent to rotating the original ConvLSTM unit 90 degrees clockwise so that the information flow is delivered in the vertical direction, so as to realize the combination with the U-Net structure. Its input is the output $\tilde{X}$ of the ECSA attention module, and $M^{l}$ is the spatial–temporal memory described. It passes vertically from the l−1th layer to the current node in the same time step, and the final hidden state $H^{l}$ also depends on the spatial memory at this time. Its forgetting gate *f*, memory gates *i* and *g*, and output gate *o* are consistent with the gate structure of the ConvLSTM unit. It has the following update formula, where ∗ is the convolution operation and ⊗ denotes the Hadamard product.

(3) $$\begin{array}{cc} \; & g=\tanh(W_{xg}*\tilde{X}+W_{hg}*H^{l-1}+W_{mg}*M^{l-1}+b_{g})\\ \; & f=\sigma (W_{xf}*\tilde{X}+W_{hf}*H^{l-1}+W_{mf}*M^{l-1}+b_{f})\\ \; & i=\sigma (W_{xi}*\tilde{X}+W_{hi}*H^{l-1}+W_{mi}*M^{l-1}+b_{i})\\ \; & M^{l}=f⊗M^{l-1}+i⊗g\\ \; & o=\sigma (W_{xo}*\tilde{X}+W_{ho}*H^{l-1}+W_{mo}*M^{l}+b_{o})\\ \; & H^{l}=o⊗\tanh\left(M^{l}\right) \end{array}$$

All states of the ConvLSTM were initialized to 0 before the first spatiotemporal state appeared.

### 3.3. Loss Function

The previous loss function of precipitation nowcasting was the Mean-Squared Error (MSE), and it can accelerate the regression of forecast data; however, it is obviously not suitable for optimizing a single measurement criterion because precipitation is a strong random and chaotic event. Therefore, we made several improvements on the basis of the MSE.

Firstly, the weighted MSE loss function in [19] was used because of the imbalance of the precipitation data samples (the first formula in (5)).

Secondly, the Critical Success Index (CSI) [34] is generally used to evaluate precipitation in the meteorological field. The case where all precipitation was greater than the set threshold was considered as 1, and the others were set as 0. Setting the TPs (prediction = 1, truth = 1), FPs (prediction = 1, truth = 0), TNs (prediction = 0, truth = 0), and FNs (prediction = 0, truth = 1), the CSI score was calculated as shown in (4)). This shows that the CSI score is a categorical indicator and the MSE is a regression loss function, so optimizing the MSE does not result in a significant improvement in the CSI score. Therefore, the binary cross-entropy loss function [35] was introduced and slightly modified into a loss function suitable for regression problems. A target value $y_{i}$ greater than the threshold was set to 1 and for the rest of the cases to 0 to obtain ${y_{i}}^{\prime }$, and the difference between the predicted value and the threshold was passed through the Sigmoid function to obtain the probability of whether the predicted value is greater than the threshold to calculate the loss function value.

(4) $$\mathrm{CSI}=\frac{\mathrm{TP}}{\mathrm{TP}+\mathrm{FP}+\mathrm{FN}}$$

Our final loss function, TLoss, is the last equation in (5), and it was experimentally proven that the improved binary cross-entropy loss function with the weight value set to 0.5 achieved the best results.

(5) $$\begin{array}{cc} \; & WMSE=\frac{1}{n}\sum_{i=1}^{n}{\left(\hat{y_{i}}-y_{i}\right)}^{2}*\left(e^{y_{i}*0.6}-0.8\right)\\ \; & \hat{y_{p}}=\sigma \left(\hat{y}-p\right)\\ \; & BCE_{p}=-\frac{1}{n}\sum_{i=1}^{n}\left({y_{i}}^{\prime }\right)*\log\hat{y_{p}}+\left(1-{y_{i}}^{\prime }\right)*\log\left(1-\hat{y_{p}}\right)\\ \; & TLoss=WMSE+0.5*(BCE_{p1}+BCE_{p2}+BCE_{p3}) \end{array}$$

In this loss function, $BCE_{p}$ denotes the binary cross-entropy loss function for each frame greater than p mm, *p*1 denotes light rainfall, *p*2 denotes moderate rainfall, and *p*3 denotes heavy rainfall.

## 4. Experiments

### 4.1. Dataset

In this paper, we used the dataset from the 2022 Jiangsu Meteorological AI Algorithm Challenge. Selecting this dataset for the experiments can make our model more widely applicable in other engineering applications.

The 2022 Jiangsu Weather AI Algorithm Challenge dataset, which has precipitation feature maps produced by Jiangsu Meteorological Station, is elemental data for the period of April–September 2019–2021. It is stored as a grayscale map, obtained by interpolating data from automatic meteorological observation stations in Jiangsu and surrounding areas to a uniform network. Interpolation objectively reflects the spatial distribution of meteorological elements and matches other meteorological data. The precipitation element is the cumulative 6 min precipitation at the automatic station, i.e., the cumulative value of precipitation observations over a 6 min period up to the current moment. The resolution of a frame is 480 × 560 pixels, ranging from 0–10 mm. In addition, the competition also provides meteorological radar data; these data were obtained from the quality control and mosaic of multiple S-band meteorological radars in Jiangsu Province. Quality control is the premise of ensuring the correct and credible meteorological observation data entering the application system such as weather forecast. The mosaic splices the fragmented regional radar monitoring map into a complete regional radar map of Jiangsu Province. Radar echo data cover the whole Jiangsu region with a horizontal resolution of 0.01° (about 1 km). It has the same spatial and temporal resolution as the precipitation element. The data range is 0–70 dBZ.

In terms of data preprocessing, first of all, because of the strong correlation between radar echo elements and precipitation rainfall magnitude and distribution, it can be seen from Table 2 that the performance of the fused data was usually stronger than that of a single precipitation factor, so the two elements were stacked at the channel level to form multi-source fused data for precipitation nowcasting. Secondly, in order to facilitate the research and due to the limitation of computing power and training cost, we downsampled the data again to 120 × 140 pixels by bilinear interpolation. Finally, in order to predict the precipitation in the next two hours, the multi-source fusion data of the past two hours were used to predict the precipitation in the next two hours, a sequence of 40 times in total. The data were divided into 27,625 groups and divided into training set and test sets with a ratio of 8:2.

### 4.2. Performance Metrics

The evaluation metrics used were the CSI [34], the Probability Of Detection (POD), and the False Alarm Rate (FAR). The CSI is the World Meteorological Organization’s scoring standard for the accuracy of quantitative precipitation forecast. The POD refers to the proportion of the predicted actual precipitation area in all actual precipitation areas. The FAR refers to the proportion of the actual non-precipitation area in the forecast precipitation area in relation to the total forecast precipitation area. The calculation of the CSI is given in Section 3. The POD and FAR scores were calculated as follows. We set the thresholds to 0.05 mm, 0.2 mm, and 0.5 mm to calculate these metrics, corresponding to light rain, light to moderate rain, and moderate to heavy rain [13]. In addition, in order to show the impact of the next 2 h rainfall nowcasting, we evaluated it in two time periods of 1 h and 2 h. The TPs, FNs, FPs, and TNs are also given in Section 3.

(6) $$\begin{array}{cc} \; & \mathrm{POD}=\frac{\mathrm{TP}}{\mathrm{TP}+\mathrm{FN}}\\ \; & \mathrm{FAR}=\frac{\mathrm{FP}}{\mathrm{TP}+\mathrm{FP}} \end{array}$$

### 4.3. Implementation Details

LSTMAtU-Net was implemented in Pytorch. We first normalized the precipitation data *M*. The normalization procedure was as follows, and the logarithmic transformation can make the precipitation data distribution as close to a Gaussian distribution as possible, thus enhancing the model fitting effect with the MSE as the loss function. We set 0.001 as the learning rate, used the Adam optimizer, and set the batch size to 20. The LSTMAtU-Net model uses the TLoss as the verification loss function. In the loss function, p1 takes 0.05, p2 takes 0.5, and p3 takes 1. Other models use the MSE loss function. All experiments were trained and tested on an NVIDIA Tesla A100 GPU.

(7) M=log(1+M)

### 4.4. Quantitative Analysis on the 2022 Jiangsu Weather AI Algorithm Challenge Dataset

We compared the LSTMAtU-Net model with other models on the 2022 Jiangsu Meteorological AI Algorithm Challenge dataset. We calculated 6 min of cumulative rainfall to evaluate the performance of these models. The average evaluation results of the 10-frame precipitation forecast in the previous hour are shown in Table 3, and the average evaluation results of the 20-frame precipitation forecast in two hours are shown in Table 4. The best performance is indicated by bolded font.

**Figure 5:**
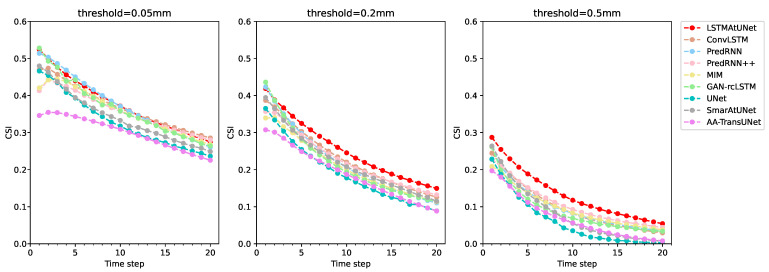
Prediction comparison of different time steps in two hours; 1 time step is 6 min.

The CSI is the main basis for comparing the performance of precipitation nowcasting methods. From the results of Table 3 and Table 4, the LSTMAtU-Net model achieved the best results on the CSI evaluation metric, and the evaluation metrics of medium and high intensity precipitation were at least 4% higher than other SOTA models. In addition, we also provide a plot of the evaluation metrics for different prediction steps in Figure 5. The red dotted lines show the prediction results of our model, and it can be seen from the figure that this model had the best prediction results in most of the prediction intervals with medium and high thresholds. This means that the LSTMAtU-Net model we propose not only improved the accuracy of precipitation nowcasting to a certain extent, but also had fair performance in long-term dependence. However, it did not achieve good performance on the FAR index, and the false alarm rate needs to be reduced. Next, we compared the evaluation results of different methods.

Comparison of the models based on the ConvRNN architecture and U-Net architecture: From Table 3 and Table 4, it can be seen that the overall CSI of the model based on the ConvRNN architecture [10,11,24,25,26] was relatively high. Compared with the overall average score of the model based on the U-Net architecture [15,16,21], the overall average CSI score of the model based on the ConvRNN architecture increased by at least 2% in the evaluation results within the first hour and second hour. The precipitation nowcasting accuracy of [15,16,21] was low, and the FAR scores were high; however, their POD scores were relatively high. The POD scores with thresholds of 0.05 mm, 0.2 mm, and 0.5 mm in the evaluation results within 2 h were at least 0.68, 0.44, and 0.16, respectively, while the POD scores in [10,11,24,25,26] were basically below these values. We believe that the U-Net architecture model can capture complex spatial changes, so their POD scores were higher. The information transmitted decayed faster with time than the model based on the ConvRNN architecture. It can be clearly seen from the third picture in Figure 5 that the gray line representing the SmaAt-U-Net model had a higher score than most of the models based on the ConvRNN architecture in the first two time steps, but the latterscore decreased faster, so their CSI scores were relatively low. The precipitation forecast accuracy of [10,11,24,25,26] was higher, and the FAR scores were lower; however, their POD scores were relatively low. This result was because the models based on the ConvRNN architecture have obvious advantages in the processing time dimension, but their ability to capture features is not strong, so their CSI scores were higher and POD scores relatively lower.

LSTMAtU-Net is a model architecture based on the U-Net architecture, so its POD score was close to or even superior to that in [15,16,21]. In the evaluation results within 2 h, the POD scores of 0.2 mm and 0.5 mm reached greater than 0.5 and 0.3, respectively. It is also equipped with a ConvLSTM unit with the vertical flow direction, which enhanced the ability to process in the time dimension. It can be seen that the LSTMAtU-Net’s score was the highest in most of the prediction intervals with medium and high thresholds in Figure 5, so it achieved the best results in the CSI evaluation metric.

**Figure 6:**
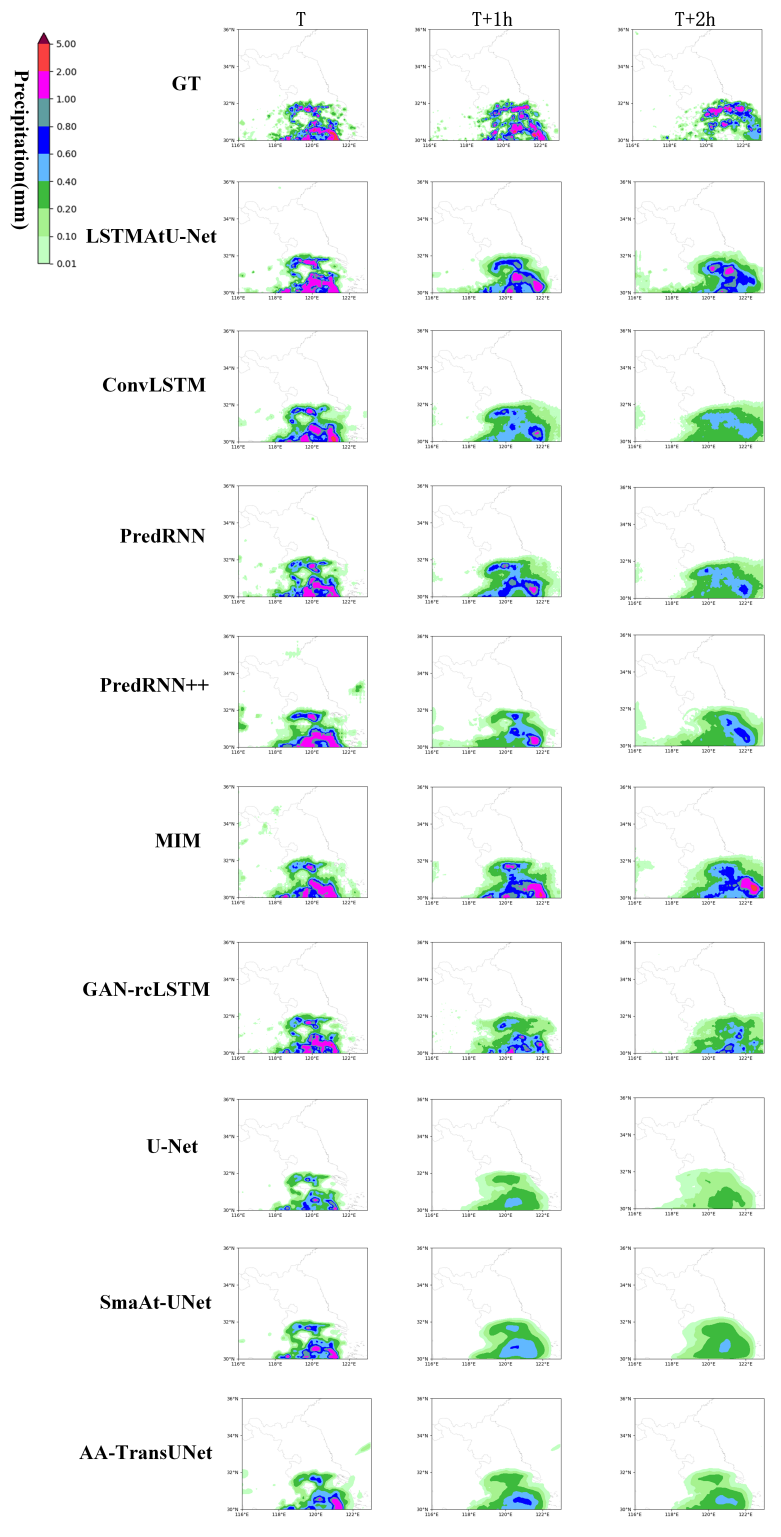
Visualization comparison with other models.

A visual example of random selection is provided in Figure 6 to further visualize the predictive power of the model. The second behavior is the prediction results of our model. Firstly, it can be seen that the image details predicted by the LSTMAtU-Net model at three time nodes were more accurate than the other models, as it alleviated the problem of the RNN and CNN prediction image details being gradually inaccurate. Secondly, our model was superior to the other models in the area and intensity of medium and high precipitation, which improved the prediction accuracy of medium and high precipitation to a certain extent. However, neither our model nor the other models wereaccurate in portraying larger value precipitation areas, and the images became more and more blurred over time; these shortcomings need to be explored in future work.

### 4.5. Ablation Experiments and Analyses

In order to fully explore the performance of the model, we used ablation experiments to explore the performance of the model architecture and the ECSA module and TLoss in the model. Since our model was based on the U-Net architecture, we used the U-Net model as the baseline model.

**Figure 7:**
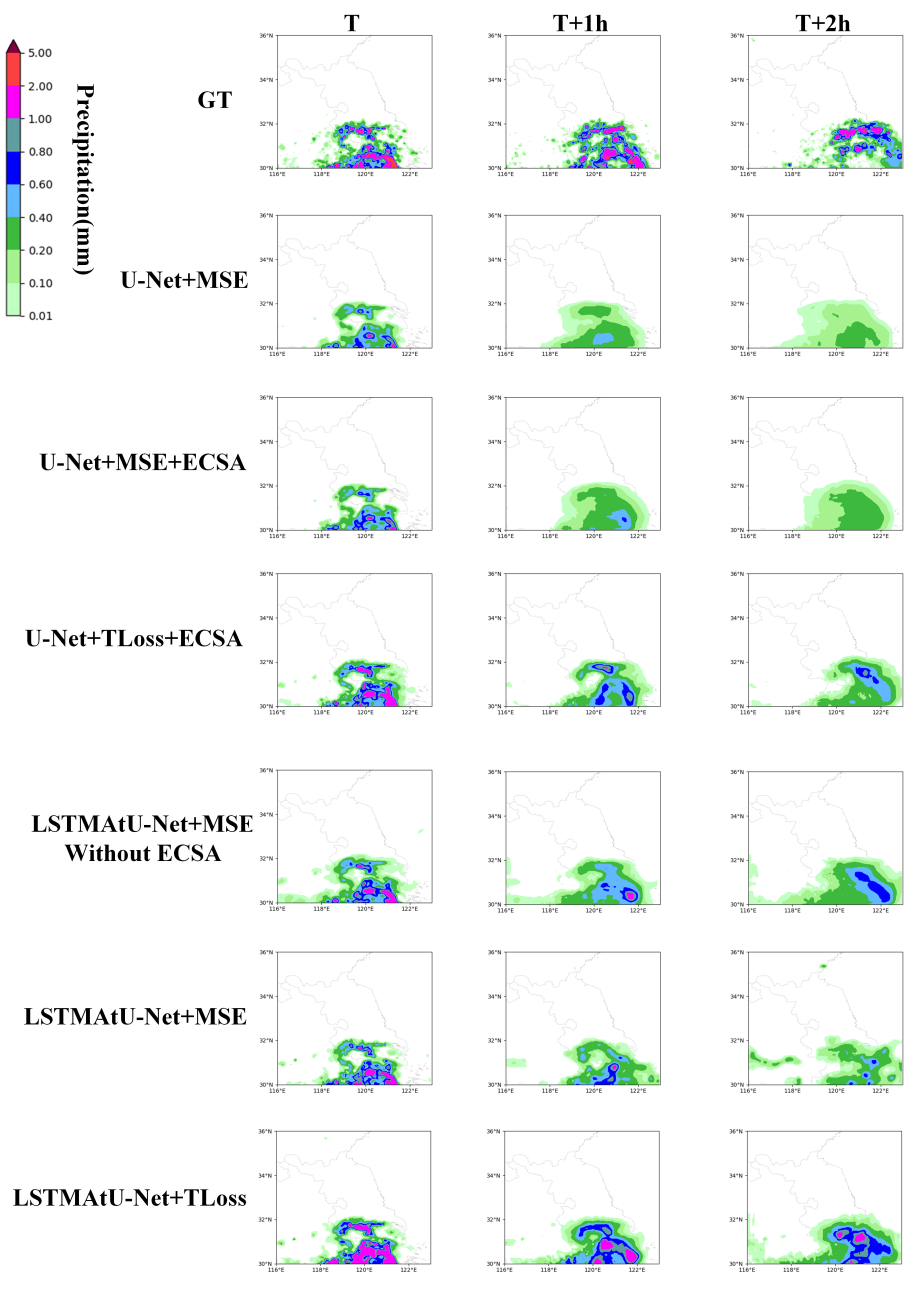
Comparison of the visualization results of the ablation experiments. The first behavior is the true value of precipitation. The second line, U-Net + MSE, means that the conventional U-Net model was used and the loss function was the MSE, and the other lines are similar. The first is the 6-minute cumulative precipitation from T to T + 6 min, the second the 6-minute cumulative precipitation from T + 60 min to T + 66 min, and the third the 6-minute cumulative precipitation from T + 114 min to T + 120 min.

Firstly, the precipitation forecast accuracy and visual images of the model with the ECSA module and the model without the ECSA module were compared to evaluate the influence of the ECSA module on the details of the forecast images. As shown in the third, fourth, sixth, and seventh lines of Table 5 and Table 6 and Figure 7, it can be seen that the ECSA module improved the accuracy of the precipitation forecast to a certain extent, and the average precipitation results for the CSI metric in 2 h were the best. In addition, in the visual image, the model with the ECSA module was more accurate than the model without the ECSA module to predict the details of the image. Therefore, it can be shown that the ECSA module can alleviate the problem of the inaccurate prediction of image details to a certain extent.

Secondly, we compared the same model using different loss functions to evaluate the impact of the TLoss on the precipitation forecast accuracy. As shown in the fourth, fifth, seventh, and eighth lines of Table 5 and Table 6 and Figure 7, it can be seen that the model using the TLoss was higher than the model using the MSE in the CSI metric, especially for the medium and high precipitation. In addition, in the image, the area and intensity of the prediction image using the TLoss function were more accurate at the medium and high thresholds. Therefore, it can be shown that the TLoss function improved the prediction accuracy of the medium and high precipitation to a certain extent.

Finally, the timing performance of the model architecture was compared. As shown in the first and fourth lines of Table 5 and Table 6 and Figure 7, it can be seen that the model with the ConvLSTM unit achieved the best CSI metric. In addition, in the image, the two-hour prediction image of the model with the ConvLSTM unit was more accurate than the U-Net model. Therefore, it can be shown that the introduction of the ConvLSTM unit improved the long-term dependence.

## 5. Conclusions

In this paper, we proposed a new precipitation nowcasting model, LSTMAtU-Net. The model combines the CNN structure, the RNN structure, and an attention mechanism. It first performs convolutional and downsampling operations on the input image sequence to extract features from the images at various scales, followed by integrating the extracted feature information with channel information and spatial structure information through the ECSA module, grasping the temporal information through the ConvLSTM unit with the vertical flow direction, then fusing the information with the previous ones at the same scale for each upsampling, and finally, fusing them through a 1 × 1 convolution to output the predicted image. Here, we validated the feasibility and correctness of the proposed model using a competition dataset and a public dataset. It can be seen from the results that our proposed model outperformed the state-of-the-art precipitation nowcasting models.

Although the proposed model showed some improvements, there were still some shortcomings. First, the forecast accuracy of medium- to high-intensity precipitation was improved, but was still low. Secondly, the accuracy of the forecast results and the clarity of the forecast images decreased with the increase of the forecast time.

The first problem was caused by the small percentage of medium- to high-intensity precipitation data and the averaging property of the convolutional operation; therefore, we need to think deeply about the setting of the weighted loss function in future studies. For the second problem, the Transformer architecture can be introduced and improved, which can effectively capture the temporal coherence of long time series. References [20,36,37,38,39,40] applied the Transformer architecture to time series forecasting with good results, while References [29,41,42,43] also used this architecture in their work on precipitation nowcasting to demonstrate the feasibility of this method. Therefore, our future research will focus on the Transformer architecture and the weighted loss function to improve the precipitation nowcasting accuracy.

## Figures and Tables

**Table 1:** Number of parameters of the compared models.

Model	Parameters
LSTMAtU-Net with SENet	32,023,892
LSTMAtU-Net with ECSA	31,948,797
LSTMAtU-Net with ECSA and DSC	18,619,421

**Table 2:** The quantitative evaluation results of the single factor and the fusion features are the average evaluation results of the 10-frame precipitation nowcasting in the previous 1 h. “r≥γ mm” refers to the skill score under the γ mm rainfall threshold within 6 min. “↑” means the higher the score, the better, while “↓” means the lower the score, the better. And the bold font indicates better performance.

Method	r≥0.05 mm	r≥0.2 mm	r≥0.5 mm
	CSI↑	CSI↑	CSI↑
U-Net (single factor)	**0.3993**	0.2432	0.0978
U-Net (fusion features)	0.3878	**0.2565**	**0.1098**
ConvLSTM (single factor)	0.4132	0.2660	0.1328
ConvLSTM (fusion features)	**0.4204**	**0.2984**	**0.1487**

**Table 3:** Comparison of average evaluation results of 10 frames of precipitation nowcasting in the first hour of different models. “r≥γ mm” refers to the skill score under the γ mm rainfall threshold within 6 min. “↑” means the higher the score, the better, while “↓” means the lower the score, the better. The best performance is indicated by bolded font.

Model	r≥0.05 mm			r≥0.2 mm			r≥0.5 mm		
	CSI↑	POD↑	FAR↓	CSI↑	POD↑	FAR↓	CSI↑	POD↑	FAR↓
U-Net [21]	0.3878	0.7607	0.5227	0.2565	0.6308	0.6313	0.1098	0.3155	0.6492
SmaAt-U-Net [15]	0.4019	**0.7795**	0.5183	0.2877	0.6042	0.5914	0.1360	0.2914	0.5214
AA-TransU-Net [16]	0.3364	0.7351	0.5741	0.2464	0.5752	0.6104	0.1152	0.3006	0.6123
ConvLSTM [10]	0.4204	0.7139	0.5059	0.2984	0.4837	0.5198	0.1487	0.2270	0.4204
PredRNN [24]	0.4427	0.6353	0.4244	0.2972	0.4297	0.4977	0.1454	0.2109	0.4834
PredRNN++ [25]	0.4021	0.6591	0.5059	0.2872	0.4502	0.4819	0.1515	0.2400	0.3993
MIM [26]	0.4094	0.6290	0.4722	0.2716	0.3925	**0.4385**	0.1377	0.2034	**0.3788**
GAN-rcLSTM [11]	0.4286	0.5917	**0.4216**	0.2886	0.4035	0.4648	0.1314	0.1840	0.4536
LSTMAtU-Net	**0.4455**	0.7766	0.4876	**0.3267**	**0.6662**	0.5859	**0.1908**	**0.4318**	0.6419

**Table 4:** Comparison of the average evaluation results of 20 frames of precipitation nowcasting in two hours of different models. “r≥γmm” refers to the skill score under the γ mm rainfall threshold within 6 min. “↑” means the higher the score, the better, while “↓” means the lower the score, the better. The best performance is indicated by bolded font.

Model	r≥0.05 mm			r≥0.2 mm			r≥0.5 mm		
	CSI↑	POD↑	FAR↓	CSI↑	POD↑	FAR↓	CSI↑	POD↑	FAR↓
U-Net [21]	0.3285	0.7220	0.5950	0.1904	0.4783	0.6827	0.0601	0.1719	0.4659
SmaAt-UNe [15]	0.3459	**0.7414**	0.5814	0.2193	0.4711	0.6152	0.0802	0.1692	**0.3704**
AA-TransU-Net [16]	0.2994	0.6836	0.6144	0.1883	0.4459	0.6508	0.0700	0.1786	0.5105
ConvLSTM [10]	0.3696	0.6873	0.5652	0.2311	0.3932	0.5557	0.0986	0.1501	0.3662
PredRNN [24]	0.3749	0.5600	0.4933	0.2221	0.3298	0.5570	0.0985	0.1447	0.5037
PredRNN++ [25]	0.3570	0.6414	0.5670	0.2260	0.3801	0.5124	0.1067	0.1760	0.3846
MIM [26]	0.3579	0.5686	0.5234	0.2095	0.3131	**0.4628**	0.0963	0.1448	0.3708
GAN-rcLSTM [11]	0.3662	0.5334	**0.4922**	0.2162	0.3189	0.5449	0.0888	0.1265	0.4934
LSTMAtU-Net	**0.3813**	0.7249	0.5542	**0.2564**	**0.5364**	0.6425	**0.1350**	**0.3025**	0.6173

**Table 5:** The average evaluation results of 10 frames of precipitation nowcasting in the first hour. “r≥γ mm” refers to the skill score under the γ mm rainfall threshold within 6 min. “↑” means the higher the score, the better, while “↓” means the lower the score, the better. The best performance is indicated by bolded font.

Model	r≥0.05 mm			r≥0.2 mm			r≥0.5 mm		
	CSI↑	POD↑	FAR↓	CSI↑	POD↑	FAR↓	CSI↑	POD↑	FAR↓
U-Net+MSE	0.3878	0.7607	0.5227	0.2565	0.6308	0.6313	0.1098	0.3155	0.6492
U-Net+ECSA+MSE	0.3983	0.7598	0.5095	0.2708	0.6645	0.6298	0.1265	0.3690	0.6764
U-Net+ECSA+TLoss	0.4449	0.6867	0.4385	0.3188	0.5828	0.5480	0.1716	0.3402	0.5820
LSTMAtU-Net without ECSA+MSE	0.4204	0.6552	**0.4184**	0.3043	0.4675	**0.5460**	0.1515	0.2270	**0.5478**
LSTMAtU-Net+MSE	0.4368	**0.7785**	0.4913	0.2997	**0.6951**	0.6260	0.1673	**0.4673**	0.7170
LSTMAtU-Net+TLoss	**0.4455**	0.7766	0.4876	**0.3267**	0.6662	0.5859	**0.1908**	0.4318	0.6419

**Table 6:** The average evaluation results of 20 frames of precipitation nowcasting in two hours. “r≥γ mm” refers to the skill score under the γ mm rainfall threshold within 6 min. “↑” means the higher the score, the better, while “↓” means the lower the score, the better. The best performance is indicated by bolded font.

Model	r≥0.05 mm			r≥0.2 mm			r≥0.5 mm		
	CSI↑	POD↑	FAR↓	CSI↑	POD↑	FAR↓	CSI↑	POD↑	FAR↓
U-Net+MSE	0.3285	0.7220	0.5950	0.1904	0.4783	0.6827	0.0601	0.1719	**0.4659**
U-Net+ECSA+MSE	0.3429	**0.7388**	0.5806	0.2109	0.5308	0.6742	0.0759	0.2191	0.5479
U-Net+ECSA+TLoss	0.3750	0.6522	0.5128	0.2407	0.4535	**0.5864**	0.1105	0.2165	0.5180
LSTMAtU-Net without ECSA+MSE	0.3654	0.5560	**0.5042**	0.2163	0.3356	0.6418	0.0967	0.1471	0.6038
LSTMAtU-Net+MSE	0.3743	0.7315	0.5582	0.2394	**0.5786**	0.6805	0.1177	**0.3224**	0.7032
LSTMAtU-Net+TLoss	**0.3813**	0.7249	0.5542	**0.2564**	0.5364	0.6425	**0.1350**	0.3025	0.6173

## Data Availability

The data presented in this study are available upon request from the corresponding author.
